# Mapping school exposure in Skopje region: A building-level dataset for seismic risk assessment

**DOI:** 10.1016/j.dib.2026.113335

**Published:** 2026-10-09

**Authors:** Lisa Jusufi, Roberta Apostolska, Dimitar Kitanovski, Eftim Zdravevski

**Affiliations:** a Institute of Earthquake Engineering and Engineering Seismology, Ss. Cyril and Methodius University in Skopje, Skopje, North Macedonia; b Faculty of Computer Science and Engineering, Ss. Cyril and Methodius University in Skopje, Skopje, North Macedonia

**Keywords:** School buildings, Exposure modelling, Seismic risk assessment, North Macedonia

## Abstract

This paper presents the Skopje School Exposure Dataset, an exposure dataset comprising 198 school buildings, including 165 primary and 33 secondary schools, located in the Skopje region of North Macedonia. Developed as part of a broader nationwide exposure model for school buildings, the dataset provides general information for each asset, including its name, address, geographical location, and administrative classification. It also includes structural characteristics classified according to the GEM Building Taxonomy v3.2, such as construction material, lateral load resisting system, ductility level, and number of storeys. Additional information covers occupancy during school hours, including pupils and staff, as well as total floor area and estimated structural replacement cost. The dataset was compiled using a bottom-up approach that integrates multiple sources, including previous research projects, governmental and cadastral records, and publicly available online sources. The Skopje region is home to one-third-of the country's population, and therefore a large share of its school buildings and pupils, making it a priority for seismic risk assessment. The school buildings in the Skopje region cover approximately 534 thousand m² of floor area (∼34% of the national total), with an estimated replacement cost of €433 million (∼34% of the national total), and a combined occupancy of around 90.7 thousand occupants (∼32% of the national total) across 17 municipalities. As the first openly available and harmonised exposure inventory of school buildings in the Skopje region, the dataset provides a transparent basis for seismic risk assessment, emergency preparedness planning, and the prioritisation of retrofit interventions for educational infrastructure.

Specifications Table

**Table:** 

Subject	Engineering & Materials science
Specific subject area	Seismic risk assessment and exposure modelling of school buildings (structural/earthquake engineering)
Type of data	Table (.cvs format) Processed data
Data collection	Compiled via a bottom-up approach integrating several sources and previously conducted research projects. Records were cross-checked to remove duplicates/closed schools. Missing structural attributes were assigned using photographic evidence and engineering judgement.
Data source location	Skopje region, North Macedonia.
Data accessibility	Repository name: Mendeley Data Data identification number: 10.17632/gjjy722fnt.1 Direct URL to data: https://data.mendeley.com/datasets/gjjy722fnt/1 Licence: Creative Commons Attribution 4.0 (CC BY 4.0)
Related research article	None

## 1 Value of the Data

• **Filling a data gap:** The dataset provides an openly available, building-by-building exposure inventory of school buildings in the Skopje region, addressing the limited availability of publicly accessible technical data on educational buildings in North Macedonia.
• **Scientific and practical applications:** The dataset can support researchers, engineers, urban planners and disaster risk management institutions in conducting seismic risk and resilience assessments of educational infrastructure. Local and municipal authorities may use the data to identify structurally vulnerable schools, prioritise seismic retrofit interventions, and support evidence-based emergency preparedness and school safety planning.
• **Interdisciplinary relevance:** By combining geographical, administrative, structural, occupancy and economic information, the dataset is relevant to fields beyond earthquake engineering, including disaster risk reduction, emergency management, educational planning, urban and regional planning and public investment prioritisation.
• **Reusability:** The building attributes are classified according to the state-of-the-art GEM Building Taxonomy v3.2 [1], supporting taxonomy mapping and the selection of appropriate fragility and vulnerability functions for subsequent seismic risk assessments. The assumptions adopted during the development of the dataset are described within this study and further documented in [2], supporting its transparent interpretation and application. The dataset can be updated as new building level information becomes available, integrated with exposure datasets for other occupancy categories, and extended to other regions. It can therefore support more comprehensive seismic risk assessments at both regional and national scales.

## 2 Background

Educational buildings are critical infrastructure assets: beyond their primary function, they frequently serve as emergency shelters and community gathering points following natural disasters, making their seismic safety a matter of both structural and social resilience. Despite this importance, no centralised, publicly accessible technical database of school buildings has previously existed for North Macedonia. Existing information is either limited to aggregate administrative statistics (number of schools, pupils, and staff, publicly available through the State Statistical Office [3] and available by request from the Ministry of Education and Sciences [4]) or fragmented across past research projects and governmental records covering only a limited number of buildings, using inconsistent classification taxonomies and spatial coverage.

To address this gap, a nationwide exposure model of school buildings in North Macedonia was developed by integrating previous research datasets, governmental records, cadastral information, and publicly available online sources [2]. This dataset presents the subset of that national model corresponding to the Skopje region (as seen in Fig. 1), the country's most densely populated statistical region and administrative centre, comprising 198 school buildings across 17 municipalities. The data were originally compiled to support the scenario-based seismic risk assessment of school buildings for the 1963 Skopje earthquake [2,5], as well as the probabilistic seismic risk assessment of school buildings of North Macedonia [6]. The exposure data are shared here independently, in full building-level detail, to enable their reuse beyond the original risk-assessment study for which they were developed.

**Fig. 1:**
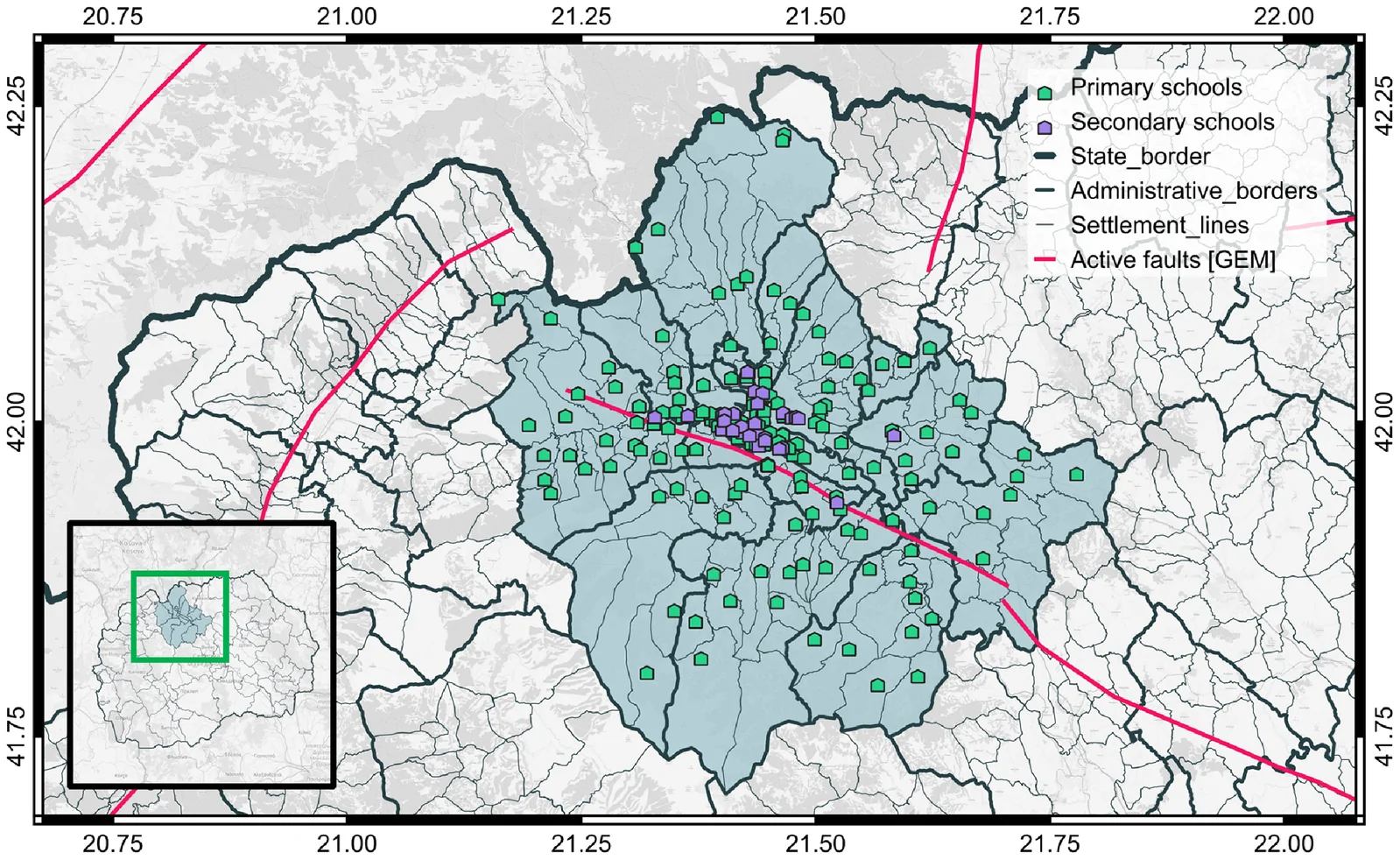
Spatial distribution of primary and secondary school buildings in Skopje region. Adopted from [5].

## 3 Data Description

The *Skopje School Exposure Dataset* consists of a single structured file (.xlsx/.csv) containing building-level records for 198 school buildings (165 primary and 33 secondary) located in the Skopje region, North Macedonia. Each row corresponds to one school building, whereas each column corresponds to one of the properties described below, grouped into three categories: general information, building classification, and occupancy and replacement cost.

*General information* (columns A-L) identifies and locates each school building, as summarised in Table 1. This includes a unique asset identifier, a region-specific ID, the names and addresses, geographic coordinates, the statistical region, school level, administrative status, municipality and its urban/rural classification, and the data source reference – an information available for all 198 buildings. Regarding the coordinates of the school buildings, they were not independently surveyed but correspond to the location already mapped for each school in Google Maps; the exact reference point used for this pre-existing placement (e.g., building entrance, footprint, or administrative address) is not known and may vary between schools. For schools not identifiable through Google Maps, the building was instead located by visual identification of its characteristic footprint on satellite imagery (school buildings typically appear as distinctive rectangular, l-shaped, or T-shaped structures accompanied by adjacent outdoor sports/playing fields) and the candidate location was then cross-checked against the AREC cadastral records; where consistent, that location was adopted as the building's coordinates. Since these coordinates are used further in risk assessment only to assign regional-scale seismic hazard parameters, positional deviations on the order of a few tens of metres do not affect the hazard values assigned to a given building. For a few rural schools where a reliable building-specific location could not be identified, the assigned coordinates instead correspond to a point at the approximate centre of the municipality rather than to the school building itself, and these records are flagged accordingly in the dataset.

**Table 1:** General information (column A-L) of the *Skopje School Exposure Dataset*.

	Description
ASSET_ID	Unique integer identifier (1 to 198)
ID	An identifier consisting of an abbreviation of the region (*Sk* for Skopje) followed by a sequential number (e.g., *Sk_1, Sk_2…*)
NAME	Name of the school
ADRESS	Adress of the school
LONGITUDE	Longitude given in decimal degrees
LATITUDE	Latitude given in decimal degrees
REGION	Skopje region (important when combining this dataset with datasets from other regions)
LEVEL	Primary of secondary school
STATUS	Central or regional school
MUNICIPALITY	One of the 17 municipalities in the Skopje region
MUNICIPALITY LEVEL	Urban or rural municipality
SOURCE	Source of data, according to [2]

The abbreviations used to describe the structural characteristics of the school buildings (columns M-P) are compatible with the GEM Building Taxonomy v3.2 [1] and are summarised in Table 2. Since this dataset is primarily created for seismic risk assessment, the chosen attributes are relevant to seismic hazard and include the structural material, lateral load resisting system (LLRS), year of construction, and number of storeys. Where information on a specific attribute was unavailable, the corresponding field was marked as “n/a”.

**Table 2:** Building classification (column M-P) of the *Skopje School Exposure Dataset*.

	Abbreviation	Description
MATERIAL	CR MCF MUR MUR+CLBRS MUR+STRUB S W	Reinforced concrete Confined masonry Unreinforced masonry Unreinforced masonry with solid bricks Unreinforced masonry with rubble stone Steel Wood
LLRS	LDUAL LFINF LWAL LFBR	Dual frame-wall system Infilled frame Wall Braced frame
YEAR	/	Year of construction / last seismic retrofit intervention
STORY	H1 H2 H3 H4	One storey above ground Two storey above ground Three storey above ground Four storey above ground

The building taxonomy strings are presented in column Q. The ductility (DUL – low ductility, DUM – moderate ductility, DNO – non-ductile) and code levels (CDL – low-code, CDM – moderate-code) were defined according to the year of construction, considering the development of seismic design regulations in North Macedonia, as described in [7]. The attributes provided separately in the dataset allow the buildings to be classified according to another taxonomy system, if necessary. There are 15 schools classified as either P-R < 20 (primary regional school with <20 pupils) or P-R > 20 (primary regional school with >20 pupils), for which information on structural characteristics was unavailable. These classification codes were therefore retained as alternative identifiers, allowing appropriate fragility and vulnerability functions to be assigned in future seismic risk assessments through a taxonomy mapping scheme.

The last section of provided data at building level are the occupancy and replacement cost data (S-Y), summarised in Table 3. The estimation of these data is presented in the following section.

**Table 3:** Occupancy and replacement cost information (column S-Y) of the *Skopje School Exposure Dataset*.

	Description
TOTAL_AREA_SQM	Gross floor area of the school building [m^2^]
TOTAL_REPL_COST_EUR	Estimated total replacement cost of the building [€]
PUPILS	Number of pupils
STAFF	Number of teaching and administrative staff
OCCUPANTS_PER_ASSET	Total occupants (number of pupils + number of staff)
OCCUPANTS_PER_ASSET_DAY	Total occupancy during the day
OCCUPANTS_PER_ASSET_NIGHT	Total occupancy during the night (for school buildings is 0)

Summary statistics for the Skopje region subset: the 198 buildings span 17 municipalities, with a combined gross floor area of 534,356 m², an estimated total replacement cost of €432,828,360, and a total school-day occupancy of 90,690 persons (81,903 pupils and 8787 staff). Thirty-two distinct GEM taxonomy strings are represented across the buildings (17 distinct structural typologies when the number of storeys is not considered), with unreinforced masonry (MUR) and reinforced concrete (CR) as the dominant structural materials.

## 4 Experimental Design, Materials and Methods

This dataset was derived from the nationwide school building exposure model of North Macedonia developed in [2], where the full data-collection strategy, taxonomy-assignment procedure, and underlying assumptions are described and justified in detail. The following provides a condensed overview of that process as applied to the 198 buildings located within the Skopje region.

No centralised, publicly accessible technical database of school buildings exists for North Macedonia. Therefore, the only available source of technical information is previous research studies and projects focused on school buildings and publicly available information on state level, i.e. implementation of a bottom-up approach for data collection. The available sources used for the development of the exposure model of school buildings in the Skopje region are the following:

• Ministry of Education and Science of North Macedonia (**MES**) [4]: Provision of administrative information, such as the total number of school facilities, their full names and addresses, level (primary/secondary), status (central/regional), number of pupils, and number of employees at the beginning of the 2022/23 academic year. This information was available upon request.
• Agency for Real Estate Cadastre of North Macedonia (**AREC**) [8]: Provision of the number of stories and net areas for all school buildings located in urban areas and for some of those located in rural areas, retrieved building-by-building through the AREC portal.
• The 2001 project of the IZIIS in Skopje, supported by UNICEF Macedonia: Physical and psychological management of earthquake related emergency situations in schools in the Republic of Macedonia (**UN-46** and **UN-47**): The primary objective of this project was to assess the state of seismic safety of the school facilities at that time and to identify the necessary measures to mitigate the potential risks of future earthquakes. In this project, the constructive and social aspects are addressed to reduce the overall risk. The findings of this project are summarized in five reports, two of which contain data on the inventory of school facilities, IZIIS Report 2001–46 [9] and IZIIS Report 2001–47 [10].
• The 2022 project of IZIIS, supported by World Bank: High-level seismic screening of the structures of municipal buildings in North Macedonia (**WB MF-EE**), a project focused on conducting a high-level seismic screening of municipal buildings, including school buildings, particularly in critical municipalities located in areas of high seismic hazard [11].
• Individual seismic risk assessment of special school buildings at the request of the municipalities or the school administration for various reasons, carried out by IZIIS (**IZIIS**).
• Online information from school websites, including reports available on their platforms (**WEB**).

Fig. 2 represents how many school buildings are considered by different data sources and their overlaps. The top bars indicate the number of schools in each unique combination of sources, while the left bars show the total number of schools recorded per source. The dot matrix below the bars highlights which sources contribute to each combination.

**Fig. 2:**
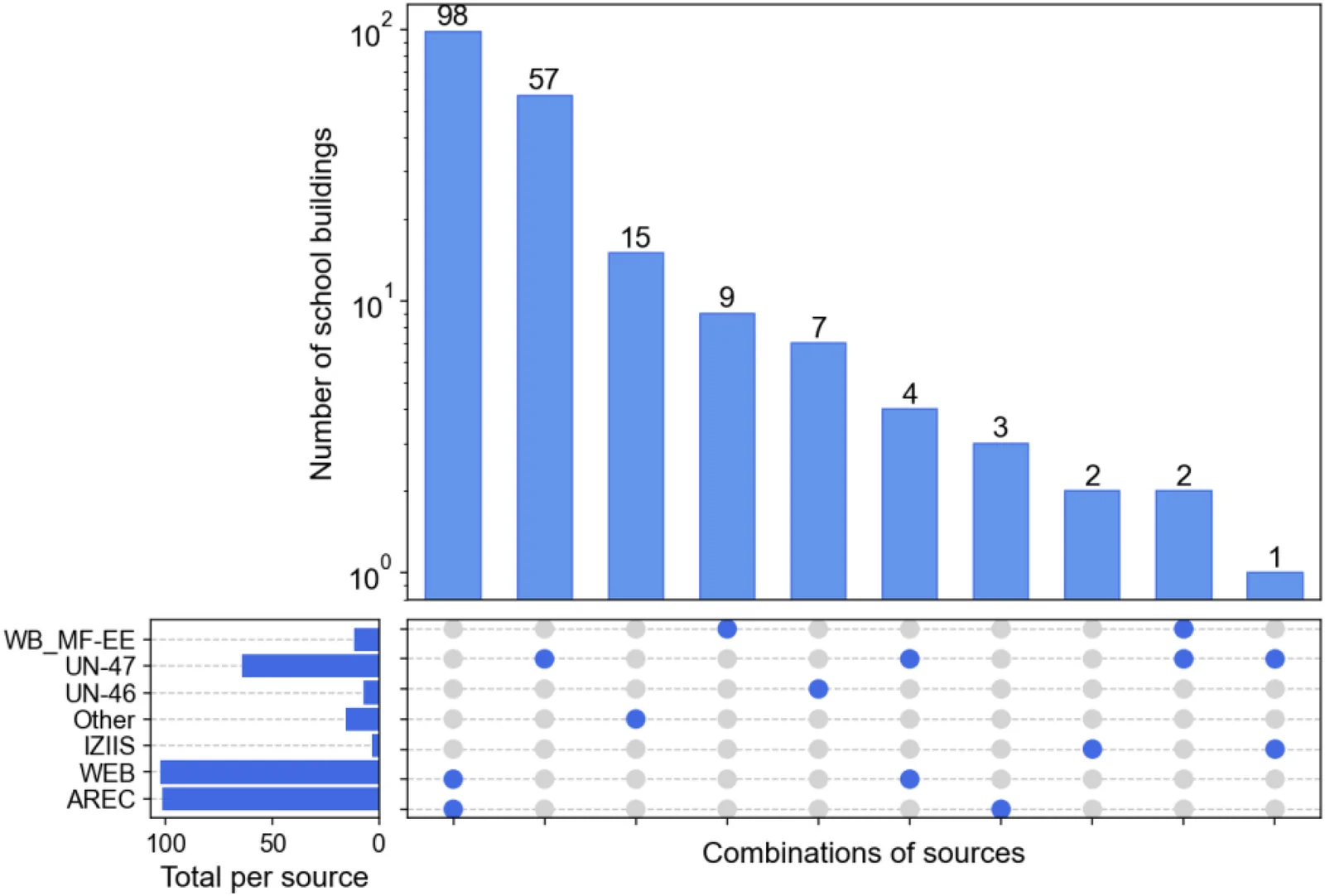
Distribution of number of buildings in Skopje region considered in various sources.

Since the consulted sources were developed for different projects and at different points in time, discrepancies between them were expected, including duplicate records and buildings that had subsequently been permanently closed or replaced. The records were therefore cross-checked and reconciled following the procedure presented in Fig. 3. Buildings officially confirmed as permanently closed were excluded, whereas schools with zero current enrolment but no formal closure notice were retained. Where reliable information was available, separate structures within the same school complex were represented individually, each identifiable through the ID_0 field (e.g., Sk_51.1 and Sk_51.2); where floor area, occupancy and replacement cost were only available at the complex level, these were divided evenly among the structural units. Incomplete building attributes were supplemented through targeted online research, including school websites and exterior and interior photographs. When the available visual evidence was insufficient for a conclusive classification, conservative assumptions were applied or the corresponding attribute was recorded as not available (N/A). The final stage involved checking the building area and calculating the replacement cost according to the procedure shown in Fig. 3.

**Fig. 3:**
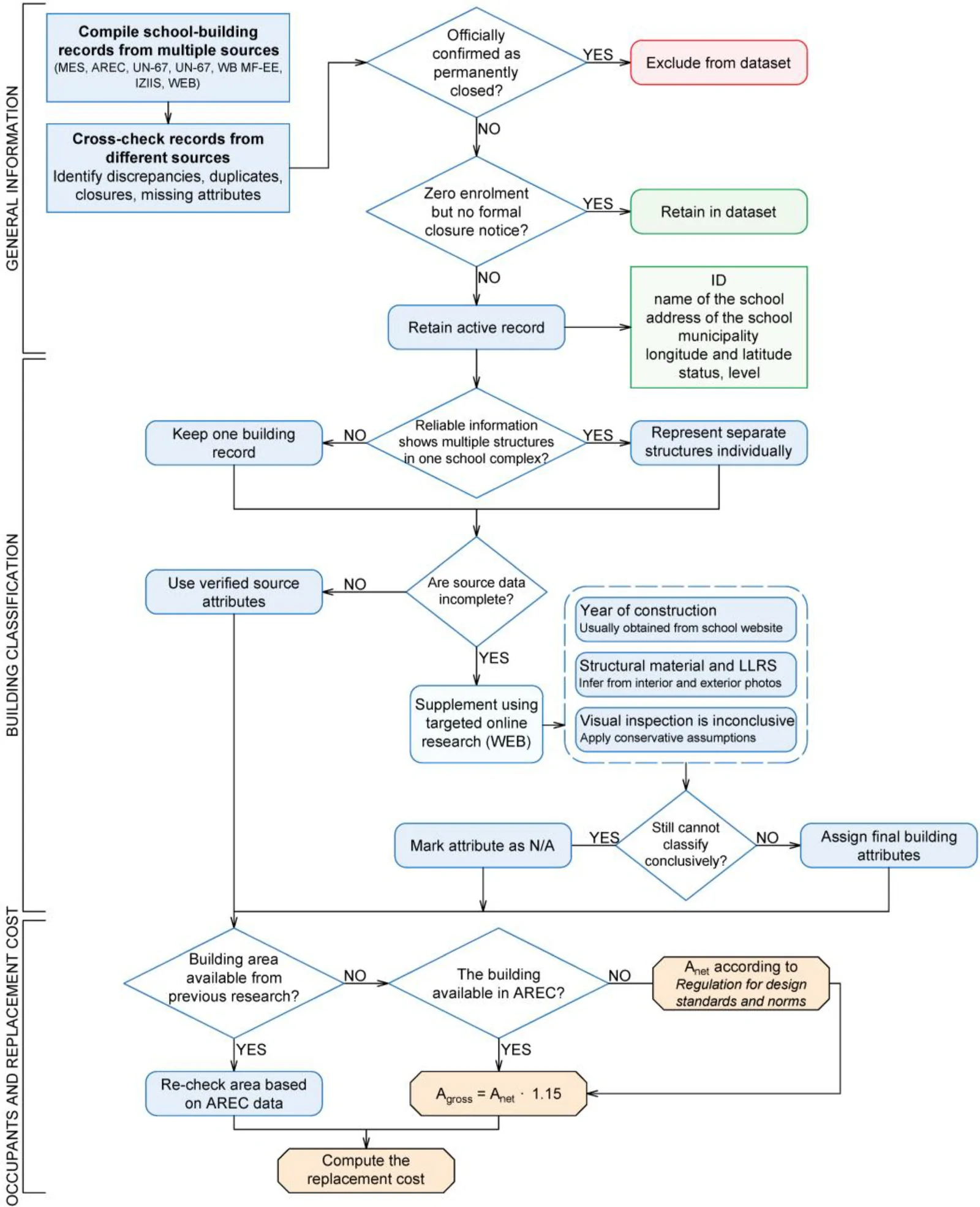
Reconciliation and data completion process for the *Skopje School Exposure Dataset*.

Fig. 4 summarises the resulting distribution of key taxonomy attributes across the 198 buildings: structural material is dominated by reinforced concrete (CR, 92 buildings), followed by confined masonry (MCF, 34) and unreinforced masonry (MUR, 34); the lateral load-resisting system is split mainly between infilled frames (LFINF, 86) and wall systems (LWAL, 90); construction period is concentrated in 1965–1981 (79 buildings), reflecting the school-building boom that followed the 1963 Skopje earthquake; and building height is predominantly one or two storeys (H1, 61; H2, 82). For each attribute, 15 buildings (7.6%) could not be conclusively classified through available records or photographic evidence and are marked as N/A in the corresponding field. In addition, 50 buildings (25.3%) have at least one structural attribute assigned via conservative assumption and engineering judgement rather than direct source data, values marked with an asterisk (*) in the dataset itself. Where a specific attribute required such an assumption, the following decision rules were applied, consistent with the approach adopted for the national exposure model [2]:

• *Missing year of construction:* assumed based on the visual characteristics of the building and the historical context of school construction in North Macedonia.
• *Missing material:* inferred by comparison with similar buildings of known typology documented in previous research or photographic evidence.
• *Missing LLRS:* assumed as a wall system for masonry buildings, and as an infilled frame for reinforced concrete buildings (dual systems where photographic evidence indicated additional structural walls).
• *Uncertain number of storeys:* primarily obtained from AREC records and verified against available photographs to resolve inconsistencies.

**Fig. 4:**
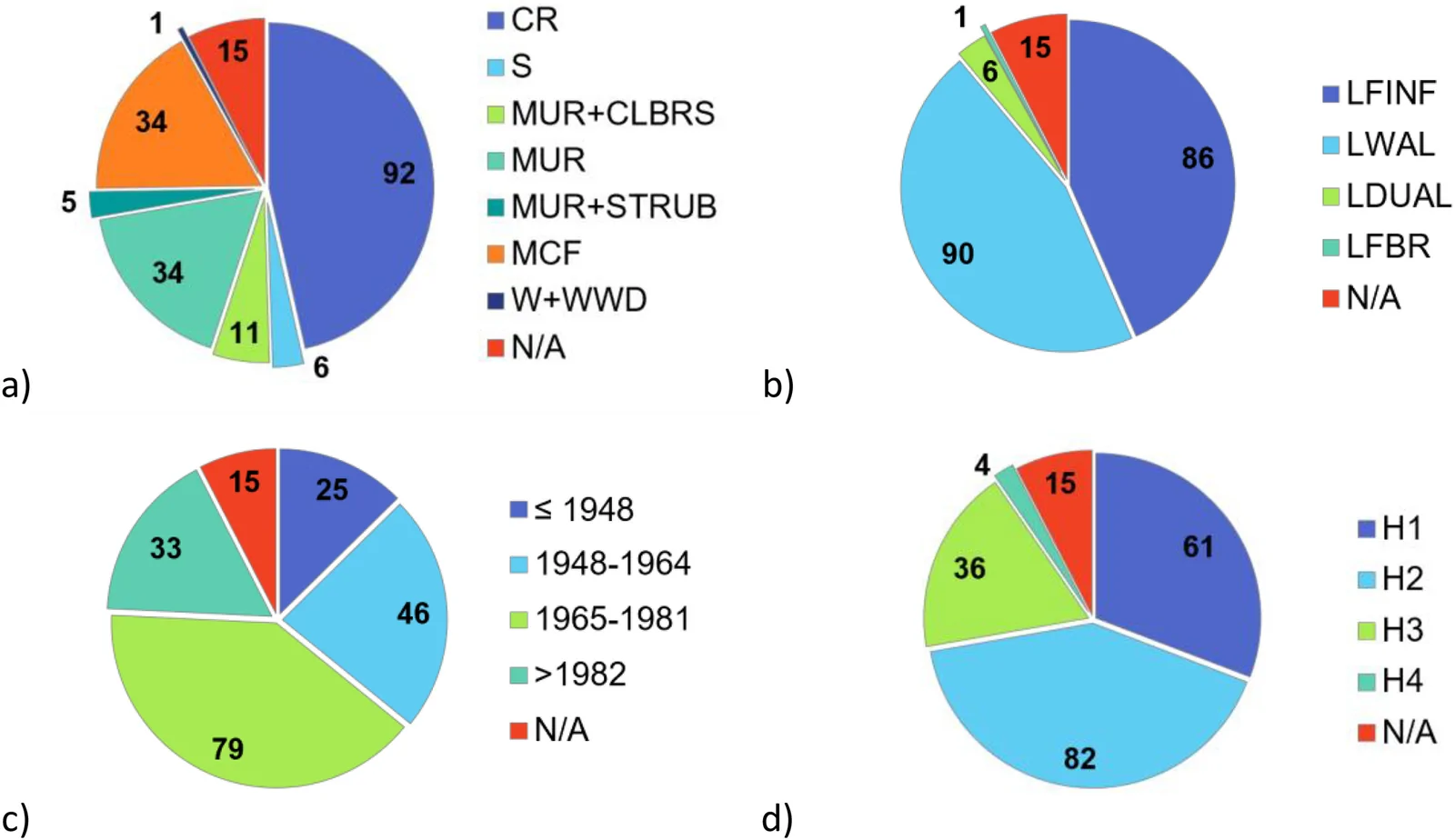
Pie charts representing the identified typologies for each main attribute: a) Material type; b) LLRS; c) Year of construction; d) Number of stories above ground.

The final piece of information, pupil and staff occupancy figures, was sourced directly from MES data. Gross floor area (A_gross_) was determined using a stepwise procedure that prioritised AREC cadastral data, applying a 15% adjustment factor for partition and perimeter walls where only net area (A_net_) was available (see Fig. 3). For the 15 buildings (7.6%) lacking geometric records (classified as “Other” in the SOURCE column of the dataset), floor area was instead estimated following the North Macedonia Regulation for Design Standards and Norms *(Official Gazette No 60/2012)*, which specifies a minimum classroom area per student, an additional corridor area scaled to the number of occupants, and a minimum sanitary area per classroom; for schools with very low occupancy, a minimum floor area of 30m^2^ was assumed to ensure a realistic building size. Replacement cost was then calculated by applying a uniform structural unit cost of 810€/m² *(Official Gazette No 54/2023)* to each building's gross floor area. Non-structural and content costs are not included.

Finally, the resulting dataset reflects a rules-based, cross-validated compilation of building-level exposure attributes for all 198 school buildings in the Skopje Region, providing a systematically documented basis for seismic risk assessment and future updates as new building-level information becomes available.

## Limitations

Where source data were incomplete, structural attributes were assigned based on conservative assumptions and engineering judgement, informed primarily by photographic evidence, and should therefore be treated as best estimates rather than verified records. Attributes assigned in this manner are flagged with an asterisk (*) in the released dataset, while coordinates assigned to the approximate centre of the corresponding municipality, rather than to the specific building, are flagged with a double asterisk (**); both symbols are defined in the accompanying database description.

The dataset has not yet been integrated into, or systematically compared with, existing exposure models such as ESRM20 or the GEM global exposure model. These models include aggregated residential, commercial and industrial exposure for North Macedonia but do not treat school buildings as a separate class, so a direct building-level comparison is not currently possible. Since the data are classified according to the GEM Taxonomy, they can be readily incorporated into such models; this integration, and comparison with school building inventories from neighbouring Balkan countries, is left for future work.

## Ethics Statement

This dataset does not involve human subjects, animal experiments, or any data collected from social media platform. All data were obtained from publicly available sources or from national institutions upon formal request.

## CRediT Author Statement

**Lisa Jusufi**: Writing – Original draft, Conceptualization, Visualization, Writing – Review & Editing. **Roberta Apostolska**: Writing – Review & Editing, Resources, Validation. **Dimitar Kitanovski**: Writing – Original draft, Conceptualization, Visualization, Writing – Review & Editing. **Eftim Zdravevski**: Writing – Review & Editing, Validation.
